# The In-Vivo Use of Superparamagnetic Iron Oxide Nanoparticles to Detect Inflammation Elicits a Cytokine Response but Does Not Aggravate Experimental Arthritis

**DOI:** 10.1371/journal.pone.0126687

**Published:** 2015-05-08

**Authors:** Eline A. Vermeij, Marije I. Koenders, Miranda B. Bennink, Lindsey A. Crowe, Lionel Maurizi, Jean-Paul Vallée, Heinrich Hofmann, Wim B. van den Berg, Peter L. E. M. van Lent, Fons A. J. van de Loo

**Affiliations:** 1 Experimental Rheumatology, Department of Rheumatology, Radboud University Medical Center, Nijmegen, the Netherlands; 2 Division of Radiology, Geneva University Hospitals, University of Geneva, Faculty of Medicine, Geneva, Switzerland; 3 Laboratory of Powder Technology, École Polytechnique Fédérale de Lausanne, Lausanne, Switzerland; University of Sao Paulo, BRAZIL

## Abstract

**Background:**

Superparamagnetic Iron Oxide Nanoparticles (SPION) are used in diagnostic imaging of a variety of different diseases. For such in-vivo application, an additional coating with a polymer, for example polyvinyl alcohol (PVA), is needed to stabilize the SPION and prevent aggregation. As the particles are foreign to the body, reaction against the SPION could occur. In this study we investigated the effects that SPION may have on experimental arthritis after intra-articular (i.a.) or intravenous (i.v.) injection.

**Methods:**

PVA-coated SPION were injected either i.a. (6 or 24 μg iron) or i.v. (100 μg or 1 mg iron) into naïve Toll-like receptor-4 deficient (TLR4-/-) or wild-type C57Bl/6 mice, or C57Bl/6 mice with antigen-induced arthritis. As control, some mice were injected with PVA or PBS. MR imaging was performed at 1 and 7 days after injection. Mice were sacrificed 2 hours and 1, 2, 7, 10 and 14 days after injection of the SPION, and RNA from synovium and liver was isolated for pro-inflammatory gene expression analysis. Serum cytokine measurements and whole knee joint histology were also performed.

**Results:**

Injection of a high dose of SPION or PVA into naïve knee joints resulted in an immediate upregulation of pro-inflammatory gene expression in the synovium. A similar gene expression profile was observed after SPION or PVA injection into knee joints of TLR4-/- mice, indicating that this effect is not due to LPS contamination. Histological analysis of the knee joints also revealed synovial inflammation after SPION injection. Two hours after i.v. injection of SPION or PVA into naïve mice, an upregulation of pro-inflammatory gene expression was detected in the liver. Administration of SPION or PVA into arthritic mice via i.a. injection did not result in an upregulation in gene expression and also no additional effects were observed on histology. MR imaging and histology showed long-term retention of SPION in the inflamed joint. However, 14 days after the injections no long-term effects were evident for gene expression, histology or serum cytokine concentrations.

**Conclusions:**

Injection of SPION, either locally or systemically, gives an acute inflammatory response. In the long term, up to 14 days after the injection, while the SPION reside in the joint, no further activating effects of SPION were observed. Hence, we conclude that SPION do not aggravate arthritis and can therefore be used safely to detect joint inflammation by MR imaging.

## Introduction

Superparamagnetic iron oxide nanoparticles (SPION) are used for imaging of anatomical, cellular and molecular changes in different diseases. Being very small particles (10–100 nm), they are taken up quickly by the mononuclear phagocyte system and can therefore travel easily to inflammatory lesions [1, 2]. Due to their unique paramagnetic properties, SPION can be visualized by Magnetic Resonance Imaging (MRI) [3–5]. SPION are already used in pre-clinical and occasionally clinical studies to detect arthritis [6, 7], as well as inflammatory bowel disease [8], central nervous system inflammatory diseases [9] and atherosclerotic lesions [10]. However, toxicity and safety profiles are still not complete and not much is known about the response to in vivo injected nanoparticles.

SPION need an additional coating with polymer to allow a longer residence time in the blood circulation and to prevent the formation of aggregates [5, 11]. Moreover, naked SPION are known to affect cell viability caused by toxic effects [12]. In case of polyvinyl alcohol (PVA)-coated particles, biocompatible PVA functionalized with amino- or carboxyl-groups are used. These groups serve for charge regulation as well as anchor points for further attachment of peptides and proteins, including antibodies, fluorescent dyes or a drug [13].

Rheumatoid arthritis is a disease characterized by chronic inflammation of articular joints, which causes irreversible cartilage and bone damage. During joint inflammation, nanoparticles will be taken up by monocytes or macrophages and then migrate through the body and to the site of inflammation. It is known that a large proportion of the nanoparticles injected intravenously (i.v.) are taken up by Kupffer cells, the macrophages in the liver [1]. However, part of the SPION will reach the target organ, the arthritic joint. The synovial lining in the joints consists of fibroblasts as well as macrophages and is therefore also capable of capturing these nanoparticles.

Several *in-vitro* and some *in-vivo* studies have shown the reaction of cells and tissues after the uptake of nanoparticles. For example, Murray *et al*. [14] reported elevated levels of the pro-inflammatory cytokines Interleukin (IL)-6 and IL-8 in the supernatant of HEK cells that were incubated with nanoparticles. Cho *et al*. [15] found lung inflammation, explained by an increase in polymorphonuclear leukocytes and lymphocytes, 24 hours after intratracheal instillation of nanoparticles. These studies showed pro-inflammatory effects after nanoparticle exposure, but were performed under healthy conditions. The question therefore arises if nanoparticles aggravate disease activity. The *in vivo* effects that nanoparticles have on the course of a disease have been studied to a lesser extent [15, 16]. Consequently, it is important to further explore the effects that nanoparticles have under disease conditions. Hence, in this study we set out to determine the effects of PVA-SPION injection, either locally or systemically, on the course of experimental arthritis. We investigated the reaction of the target organ, the synovial tissue, as well as the main systemic organ, the liver, after SPION injection in both arthritic and non-arthritic animals. Gene expression profiles, blood serum cytokine levels and histological joint inflammation were studied. We showed that injection of SPION resulted in an acute and short-lasting inflammatory response in naïve mice, but no adverse effects were observed on the course of chronic antigen-induced arthritis.

## Materials and Methods

### SPION production and characterization

#### SPION production

SPION were synthesized following a co-precipitation method in order to obtain a maghemite phase (γ-Fe_2_O_3_) suspension [17]. Briefly, a 1:2 molar ratio of 1.5 L Fe^2+^:Fe^3+^ solution (0.064 moles from FeCl_2_ and 0.128 moles from FeCl_3_) were mixed with 120 ml of ammonia solution (NH_4_OH at 25%). 10 minutes after reaction, the black suspension obtained was magnetically washed with deionized water until the pH reached 7. The SPION were then oxidized from magnetite (Fe_3_O_4_) to maghemite phase (γ-Fe_2_O_3_) by redispersion in 160 ml of nitric acid (HNO_3_ at 2 M) and 240 ml of ferric nitrate (Fe(NO_3_)_3_ at 0.35 M) under reflux for 90 minutes. The suspension was sedimented onto a magnet to remove the excess of reactant, was redispersed in DI water and dialyzed (MWCO 12–14 kDa cellulose membrane tubing) against HNO_3_ (0.01 M) for 3 days, changing the solution every 12 hours. The suspension was finally centrifuged 15 minutes at 30,000 g keeping only the supernatant. The final suspension, called naked SPION, was at 10 mg_Fe_.ml^-1^ and at approximately pH 2. The naked SPION were surface modified using a mixture of PVA-OH and amino functionalized PVA as described previously [13, 18]. A mixture of 10 volumes of naked SPION at 10 mg_Fe_.ml^-1^, 9 volumes of PVA-OH at 100 mg.ml^-1^ (Mowiol 3–85 from Kuraray Europe GmbH) and 1 volume of amino-PVA at 20 mg.ml^-1^ (M12 from Erkol) was prepared. The suspension of PVA-SPION was stored at 4°C at least one week before further use.

#### SPION characterizations

The crystallite’s mean diameter (d_TEM_) was measured by counting and averaging 400 crystallite diameters observed on Transmission Electron Microscopy (TEM CM12: FEI Co. Philips Electron Optics, Zürich, Switzerland at 125.000 X magnification and 120 kV). Hydrodynamic diameter (d_H_) as well as Zeta potential were measured on PCS apparatus (Zeta-Pals from Brookhaven: Laborchemie GES.M.B.H., Vienna, Austria) at pH 4.5 for naked SPION and 5.7 for PVA-SPION and with a refractive index of 2.42. SPION concentrations (c_Fe_ in mg_Fe_.ml^-1^) were measured using a magneto-susceptometer MS3 from Bartington, using the method described elsewhere [18]. The mass concentration of PVA versus iron (mg_PVA_/mg_Fe_) was calculated using the loss of mass (%_TGA_) measured by thermogravimetric analyses (Mettler Toledo 851e) from 30 to 800°C at 10°C.min^-1^ under 30 ml.min^-1^ of air and the c_Fe_ measured above.

### Ethics statement

All in-vivo studies complied with national legislation and were approved by local authorities (Animal Ethics Committee, Radboud University Nijmegen, Permit numbers: 2013–035, 2013–162 and 2013–193) for the care and use of animals with related codes of practice.

### Animals

Female, 12 week old C57Bl/6J mice were obtained from Janvier (Le Genest Saint Isle, France). Male and female, 8–16 week old TLR4^-^/^-^ mice in C57Bl/6J background were bred in house. All mice were housed in filter-top cages with food and water supplied *ad libitum*. The mice were provided with an in house shelter of a specially designed transparent igloo and were offered cotton wool as nesting material. Mice were i.a. injected with either 6 μg Fe/knee (low dose) or 24 μg Fe/knee (high dose) in 6 μl total volume. Mice injected i.v. received either 3.3 mg Fe/kg (low dose) or 33.3 mg Fe/kg (high dose) in 200 μl. Control injections (i.a. and i.v.) were performed with equal volumes of PBS. Mice were euthanized by cervical dislocation at different time points after injection of the SPION.

### Induction of experimental arthritis

Mice were immunized with 100 μg of mBSA (Sigma), emulsified in 100 μl Freund's complete adjuvant (Difco Laboratories, Detroit, MI). Injections were divided over both flanks. Heat-inactivated *Bordetella pertussis* (RIVM, Bilthoven, The Netherlands) was administered intraperitoneally as an additional adjuvant. Two subcutaneous booster injections in the neck region with (in total) 50 μg mBSA/Freund's complete adjuvant were given 1 week after the initial immunization. Three weeks after these injections, primary AIA was induced by injecting 60 μg of mBSA in 6 μl of PBS into the right knee joint, resulting in chronic arthritis.

### RNA isolation and quantitative PCR analysis

Single synovial tissue biopsies were isolated from the lateral and medial side of the knee joint using a 3 mm biopsy punch (Stiefel, Wachtersbach, Germany) and pooled as one sample. Total RNA was extracted from the tissue homogenates using TRI reagent (Sigma) according to manufacturer’s protocol. Isolated RNA was treated with DNAse followed by reverse transcription of 1μg RNA into cDNA using Moloney murine leukemia virus reverse transcriptase 0.5μg/μl oligo(dT) primers, and 12.5mM dNTPs (Invitrogen). Quantitative real-time PCR was performed using the StepOnePlus sequence detection system (Applied biosystems). PCR was performed in a total reaction volume of 12.5 μl consisting of appropriate cDNA, 5 μM forward and reverse primer and SYBR green PCR master mix (Applied biosystems). The PCR protocol consisted of 2 min at 50°C and 10 min at 95°C, followed by 40 cycles of 15 sec at 95°C and 1 min at 60°C. The cycle threshold value (Ct) of genes of interest was compared to the Ct of reference gene glyceraldehyde-3-phophate dehydrogenase (GAPDH) (delta Ct).

### Serum cytokine measurements

Keratinocyte chemoattractant (KC) and IL-6 levels in the blood serum were determined on a Luminex-100 system (Luminex Corp) using a bead-based multiplex immunoassay (Milliplex, Merck Millipore). Data analysis was performed with Bio-Plex Manager software (Bio-Rad Laboratories).

### MR-imaging

MR imaging was carried out on a Siemens 3T Tim Trio clinical scanner, with 42 samples per group scanned in a single acquisition arranged in the manufacturer’s wrist coil. A T1-weighted gradient echo sequence was used to acquire these ex-vivo images of the mouse knees. Coronal slices were aligned with the knee joint from a 3D isotropic resolution acquisition of 310μm, with parameters TR/TE 14.3/5.9ms and flip angle 12°. Hypo-intense signal regions were identified as resulting from accumulation of SPION particles.

### Histology

Knee joints were isolated, fixed in phosphate buffered 4% formaldehyde, decalcified with 5% formic acid, dehydrated and embedded in paraffin. Serial sections of 7μm were cut and stained with Haematoxylin/Eosin to study inflammatory cell influx. These histopathological changes were scored on an arbitrary scale ranging from 0–3 per joint. Inflammation was graded from 0 to 3 as follows: 0 = no inflammatory cells in joint cavity and a healthy, un-inflamed appearance of the synovium, 1 = a few inflammatory cells in joint cavity and synovium, 2 = joint cavity and synovium partly filled with inflammatory cells, 3 = joint cavity and synovium totally filled with inflammatory cells. Proteoglycan depletion was graded 0 to 3 as follows: 0 = no loss of safranin-O red staining of glycosaminoglycans in the cartilage, 1 = some loss of red staining, 2 = moderate loss of red staining, 3 = total loss of red staining in the upper metabolically active layers of the cartilage (above the tidemark of the deeper calcified cartilage layer).

### Statistics

Statistical differences between experimental groups were tested using the Mann-Whitney *U*-test, unless stated otherwise (Graphpad Prism V4.0). *P* values less than 0.05 were considered significant. Results are expressed as the mean ± SEM.

## Results

### SPION characterization

**Fig 1:**
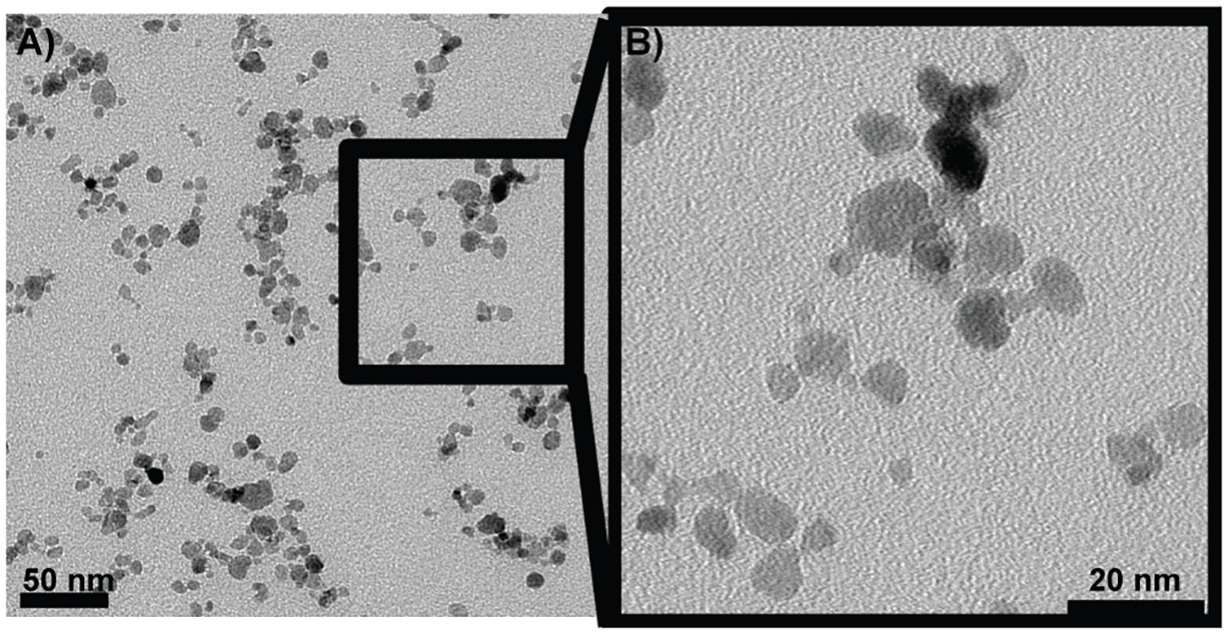
Transmission Electron Microscopy of SPION. (A) magnification 45000x and (B) magnification 125000x.

The crystallite size of the SPION used in our studies is around 8 nm (Fig 1). The hydrodynamic diameter of the naked SPION is 14 nm and increases to 45nm when covered with a PVA layer, proving the effective adsorption of PVA onto naked SPION surface and a small aggregation of the naked SPION in a PVA-hydrogel cluster [17]. The PVA coating was also shown by the loss of mass observed in thermogravimetric analyses (TGA) (PVA/Fe ratio, Table 1). The Zeta potentials were positive for naked and PVA coated particles at +26 mV and +17 mV, respectively. The PVA-SPION concentrations are at 4.7 mgFe.ml^-1^.

**Table 1:** Characterization of naked SPION and PVA coated SPION.

Nanoparticles	d_TEM_ (nm)	d_H_ (nm) [pH]	Zeta potential (mV) [pH]	PVA/Fe ratio (mg_PVA_/mg_Fe_)	c_Fe_ (mg_Fe_.ml^-1^)
Naked-SPION	7.8 ± 2	14 ± 2 [4.5]	+26 ± 2 [4.5]	0	9.4
PVA-SPION	7.8 ± 2	45 ± 3 [5.7]	+17 ± 2 [5.7]	9	4.7

d_TEM_: crystallite’s diameter; d_H_: hydrodynamic diameter; c_Fe_: concentration of nanoparticles in mg_Fe_.ml^-1^ measured in TGA.

### SPION migrate to, and reside in, articular joints after i.v. or i.a. injection

**Fig 2:**
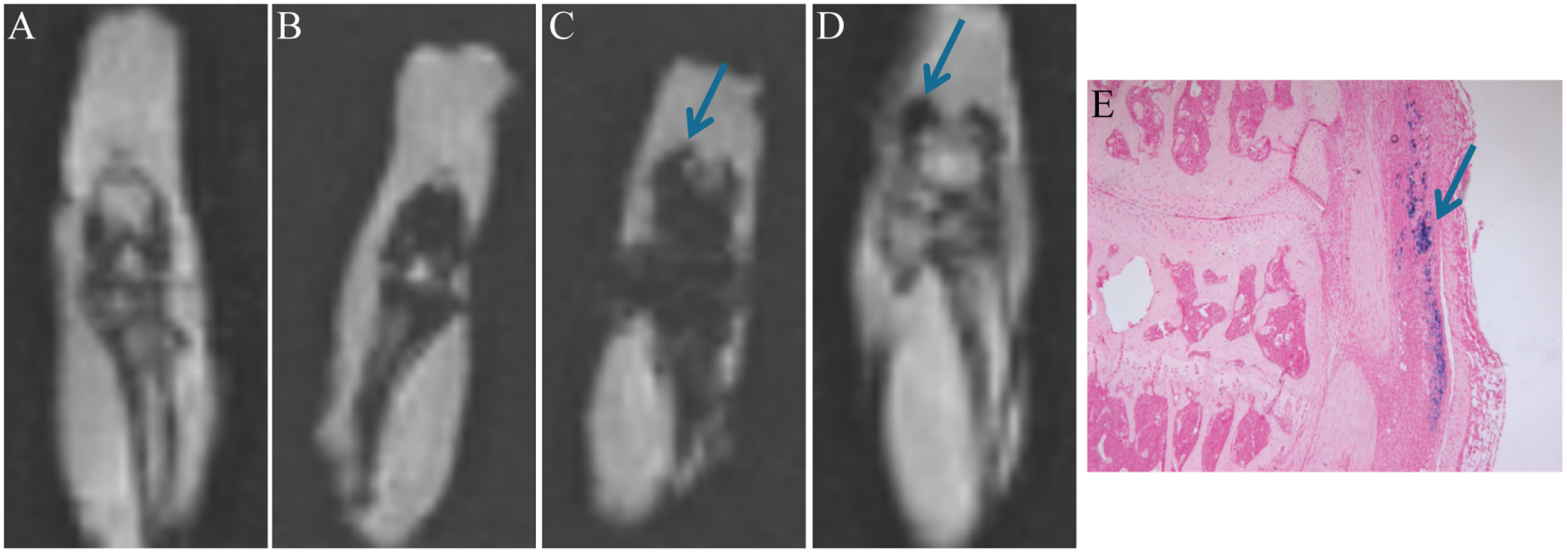
MR-imaging and Prussian blue histology visualizes SPION content in knee joints of mice. Coronal MR images of knee joints *ex vivo*. T1-weighted gradient echo sequence, 3D isotropic resolution of 310μm, TR/TE 14.3/5.9ms and flip angle 12°. Blue arrows indicate hypointense signal in the region of SPION uptake in synovial tissue. (A) Control knee joint with AIA and no SPION. (B) Control knee joint without AIA and with i.v. injected SPION; signal from SPION detected in bone marrow, but not in synovium. (C) AIA knee joint i.v. injected with SPION; signal from SPION detected in bone marrow and synovial tissue. (D) AIA knee joint injected i.a. with SPION; signal from SPION detected in synovial tissue. (E) Prussian blue staining of histological slide of AIA knee joint with SPION injected i.a. Blue arrow indicates Prussian blue staining of SPION taken up by cells of the synovium. Magnification 100x.

After i.v. injection SPION are distributed to the synovial tissue of the inflamed, arthritic joint, but not to a non-inflamed joint, as was visualized using MR imaging (Fig 2a–2c). No MRI signal was detected in an AIA joint without SPION (Fig 2a). SPION however were also taken up in the bone marrow of the femur and tibia of both arthritic and non-arthritic joints (Fig 2b and 2c). The i.v. injected SPION resided in the inflamed synovial tissue for at least 7 days, evidenced by MR imaging (Fig 2c). After i.a. injection, SPION remained in the joint where they were taken up by cells from the synovial lining (Fig 2d). SPION resided for at least 14 days in the joint, in either an inflammatory or a non-inflammatory environment. This was shown using the Prussian blue staining (Fig 2e), where blue-coloured cells were still visible in the synovial lining 14 days after SPION injection.

### SPION provoke an inflammatory response in naïve knee joints

**Fig 3:**
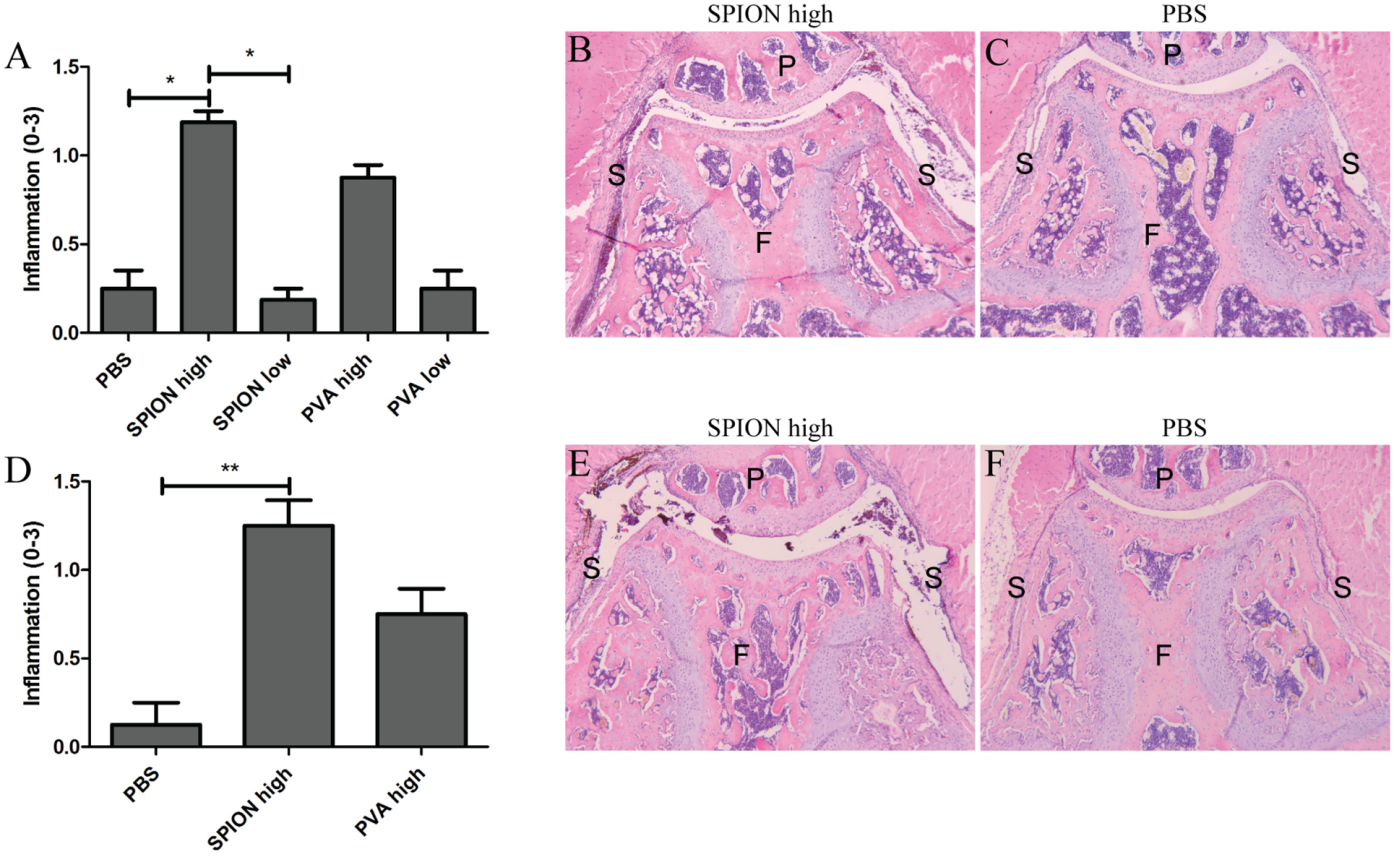
Histological analysis shows more inflammation in knee joints 1 day after i.a. injection of SPION. Histological scoring of inflammation in knee joints of naïve C57Bl/6 mice (A) or naïve TLR4-/- mice (D), 1 day after i.a. injection of SPION. Significantly more inflammation is seen in knee joints injected with the high concentration of SPION in both C57Bl/6 mice (B) and TLR4-/- mice (E) compared to PBS injected knee joints of C57Bl/6 mice (C) or TLR4-/- mice (F). Magnification 50x. F = femur, P = patella, S = synovium. n = 4 mice per group, * p<0.05, ** p<0.01 when using a one-way ANOVA.

After local (i.a.) and systemic (i.v.) administration, a difference between high or a low dose injections of SPION or PVA was found; the high dose elicited an inflammatory response whereas the low dose mostly showed no significant differences. Therefore, further results are only described for the high doses of SPION or PVA. SPION injected locally into naïve knee joints of C57Bl/6 mice caused a significant upregulation of the pro-inflammatory genes IL-1β, IL-6 and KC 2 hours after SPION injection, when compared to knee joints injected with PBS (Table 2). Injection of PVA alone also provoked an inflammatory response. After i.v. or i.a. injections of SPION in Toll-like receptor-4 knockout (TLR4-/-) mice a similar upregulation of pro-inflammatory genes was seen compared to C57Bl/6,. Hence, upregulation of pro-inflammatory genes after SPION injection was not due to Lipopolysaccharide (LPS) contamination (Table 2). Histological analysis demonstrated that inflammatory cell influx was significantly higher one day after injection of SPION, in both C57Bl/6 and TLR4-/- mice, compared to PBS control group (Fig 3) In contrast, inflammatory cell influx was not significantly raised after PVA injections. However, differences between C57Bl/6, TLR4-/- and the PBS control group were no longer seen two days after SPION injection (data not shown). Proteoglycan depletion in the cartilage was absent at all time points. When SPION were injected systemically, an upregulation of the IL-1β gene in the synovium was detected (Table 2), but no signs of joint pathology were observed on histology (data not shown). When studying the effects on gene expressions in the liver, a significant upregulation of the pro-inflammatory genes IL-1β and Tumor Necrosis Factor-α (TNFα), and of the acute phase protein Serum amyloid a-1 (Saa1) was found after i.v. injection of SPION or PVA alone (Table 3). Local injections of nanoparticles or its coating did not provoke such an inflammatory response.

**Table 2:** Gene expression in synovial tissue of naïve mice two hours after SPION or PVA injection.

		C57Bl/6				TLR4-/-
		High dose PVA-SPION	Low dose PVA-SPION	High dose PVA	Low dose PVA	High dose PVA-SPION
**TNFα**	**i.a.**	1.6 ± 0.5	0.2 ± 1.0	3.5 ± 0.2**	1.0 ± 0.3	1.2 ± 0.4
	**i.v.**	1.4 ± 0.4	0.2 ± 0.6	3.0 ± 0.3*	0.1 ± 0.1	1.9 ± 0.4
**IL-1β**	**i.a.**	5.1 ± 0.9*	1.9 ± 1.9	5.2 ± 0.6*	2.7 ± 0.4	3.4 ± 0.9*
	**i.v.**	4.3 ± 0.3*	2.6 ± 1.4	4.5 ± 0.2**	1.5 ± 0.6	4.3 ± 0.6
**IL-6**	**i.a.**	6.4 ± 0.7*	3.3 ± 1.3	7.3 ± 0.6**	2.7 ± 0.3	4.2 ± 1.0*
	**i.v.**	3.6 ± 0.6	0.6 ± 0.6	6.8 ± 0.1**	0.3 ± 0.3	0.0 ± 0.3
**KC**	**i.a.**	5.1 ± 0.7*	2.6 ± 1.4	5.9 ± 1.0**	2.1 ± 2.1	3.7 ± 0.8*
	**i.v.**	4.4 ± 0.7	0.6 ± 1.1	7.4 ± 0.2*	-0.6 ± 0.3	3.0 ± 0.4

Gene expression in synovial tissue of naïve C57Bl/6 and TLR4-/- mice, 2 hours after injection of PVA-SPIONs or PVA alone. Depicted as mean ± SEM, ddCt corrected for PBS injections and GAPDH. n = 4 mice per group,

* = p<0.05,

** = p<0.01 when using a one-way ANOVA (C57Bl/6) or Mann-Whitney U test (TLR4-/-) compared to PBS injected.

**Table 3:** Gene expression in the liver of naïve mice two hours after SPION or PVA injection.

		High dose PVA-SPION	Low dose PVA-SPION	High dose PVA	Low dose PVA
**TNFα**	**i.a.**	-0.1 ± 0.4	0.3 ± 1.7	0.3 ± 0.6	-0.2 ± 0.0
	**i.v.**	5.5 ± 0.7**	1.0 ± 0.1	6.1 ± 0.3**	1.3 ± 0.9
**IL-1β**	**i.a.**	0.0 ± 0.4	0.7 ± 0.5	0.3 ± 0.2	-0.6 ± 0.7
	**i.v.**	5.1 ± 0.5**	1.1 ± 0.6	5.9 ± 0.7**	1.9 ± 0.7
**Saa1**	**i.a.**	1.4 ± 0.5	0.2 ± 0.1	1.0 ± 0.5	1.0 ± 0.2
	**i.v.**	6.1 ± 0.8***	1.1 ± 0.1	6.6 ± 0.2***	1.3 ± 0.1

Gene expression in the liver of naïve C57Bl/6 mice, 2 hours after injection of PVA-SPIONs or PVA alone. Depicted as mean ± SEM, ddCt corrected for PBS injections and GAPDH. n = 3 mice per group,

** = p<0.01,

*** = p<0.0001 when using a one-way ANOVA compared to PBS injected.

### Injection of SPION does not aggravate arthritis

**Fig 4:**
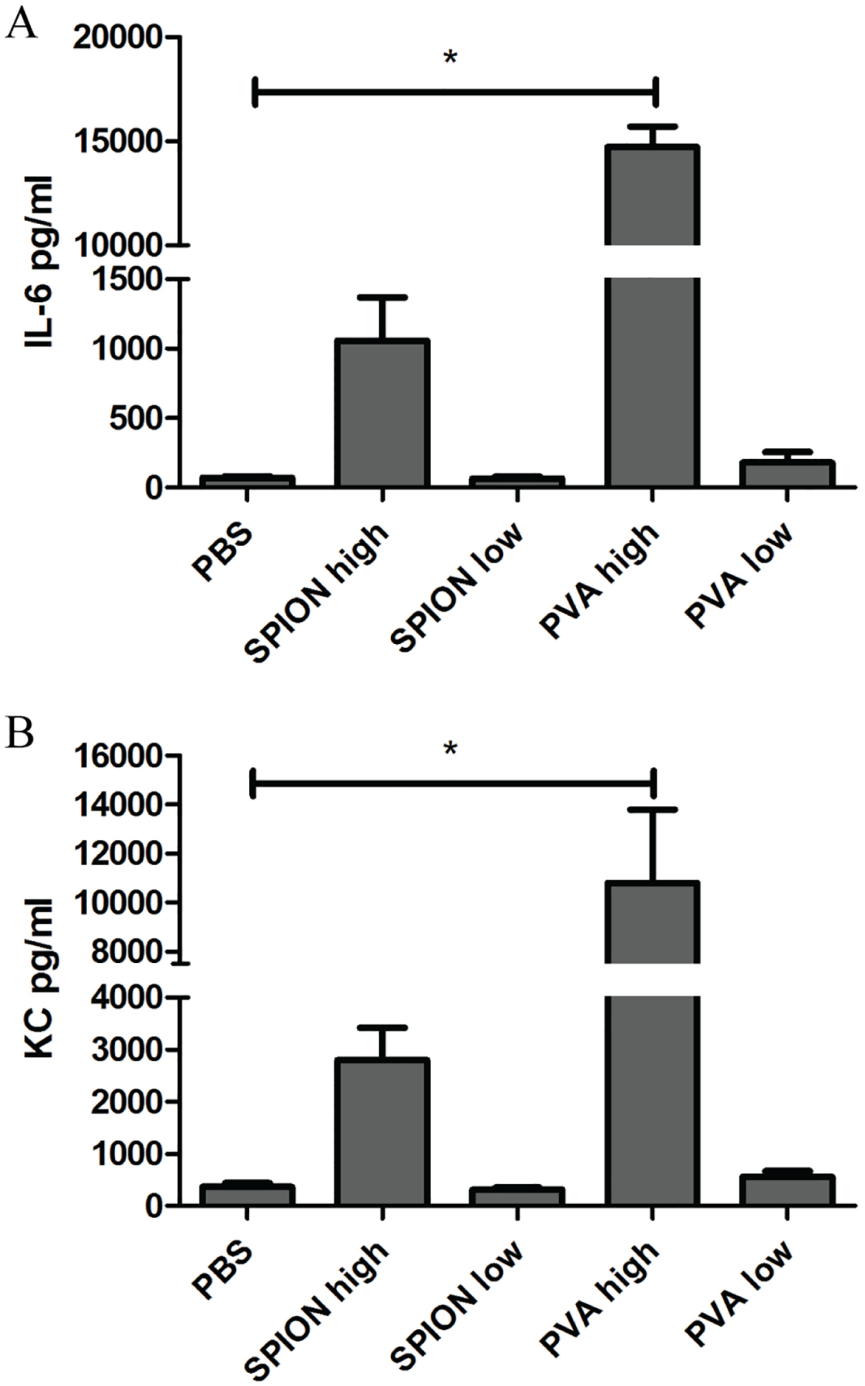
Serum cytokine concentrations are significantly elevated after i.v. injection of a high concentration of PVA. Serum cytokine concentration of IL-6 (A) and KC (B), 2 hours after i.v. injection of SPION or PVA in arthritic mice. The high concentration of PVA alone provoked a significant higher concentration of IL-6 and KC, injection of SPION gave a trend towards a higher concentration compared to PBS injections. n = 3 mice per group, * p<0.05 when using a one-way ANOVA.

Intra-articular injection of SPION during experimental arthritis caused a minor upregulation of TNFα and IL-1β gene expression in the synovium at 2 days after injection, which was on the 5^th^ day of the AIA model (Table 4). No differences were found in serum protein concentrations of IL-6 nor KC at all days, nor was any increase of inflammatory cell influx or cartilage proteoglycan depletion observed on histology (data not shown). Local injection of PVA into the arthritic joint gave no inflammatory response at all. Almost the same results were seen after i.v. SPION or PVA injection in arthritic mice; i.e. no upregulation of pro-inflammatory genes in the synovium and no enhanced joint pathology on histology. In the liver, on the other hand, i.v. injected SPION or PVA induced a significant upregulation of TNFα and IL-1β gene expression after two hours, on day 3 of the AIA model. (Table 5). After i.a. injection of SPION or PVA alone no effects were observed on gene expressions in the liver. Acute phase protein gene expression of Saa1 showed a trend toward upregulation. In the serum, IL-6 and KC protein concentrations showed a trend to be higher 2 hours after i.v. SPION injection, but only PVA alone caused a significant upregulation (Fig 4). This effect was completely absent at 1 and 2 days after SPION injections, corresponding to day 4 and 5 of the AIA model (data not shown).

**Table 4:** Gene expression in synovial tissue of arthritic mice two days after SPION or PVA injection.

		High dose PVA-SPION	Low dose PVA-SPION	High dose PVA	Low dose PVA
**TNFα**	**i.a.**	2.2 ± 0.2*	2.0 ± 0.2*	0.5 ± 0.5	0.6 ± 0.3
	**i.v.**	0.6 ± 0.7	1.1 ± 0.3	0.0 ± 0.6	-0.2 ± 0.5
**IL-1β**	**i.a.**	2.9 ± 0.4*	2.5 ± 0.1*	1.1 ± 0.5	1.0 ± 0.5
	**i.v.**	-0.3 ± 0.6	0.8 ± 0.1	-0.1 ± 0.4	-0.7 ± 0.8
**IL-6**	**i.a.**	1.8 ± 0.5	2.2 ± 0.3	1.0 ± 0.8	0.5 ± 0.7
	**i.v.**	0.0 ± 0.5	1.9 ± 0.4	0.3 ± 0.7	-0.3 ± 1.0
**KC**	**i.a.**	1.7 ± 0.5	1.3 ± 0.4	1.0 ± 0.6	0.6 ± 0.5
	**i.v.**	-0.4 ± 0.5	1.1 ± 0.2	0.3 ± 0.6	-0.6 ± 0.8

Gene expression in synovial tissue of arthritic C57Bl/6 mice at day 5 of the AIA model, 2 days after injection of PVA-SPIONs or PVA alone. Depicted as mean ± SEM, ddCt corrected for PBS injections and GAPDH. n = 4 mice per group,

* = p<0.05when using a one-way ANOVA compared to PBS injected.

**Table 5:** Gene expression in the liver of arthritic mice two hours after SPION or PVA injection.

		High dose PVA-SPION	Low dose PVA-SPION	High dose PVA	Low dose PVA
**TNFα**	**i.a.**	0.1 ± 0.4	0.4 ± 1.6	0.8 ± 0.2	-0.1 ± 0.5
	**i.v.**	4.8 ± 0.2***	0.6 ± 0.7	4.4 ± 0.3***	2.1 ± 0.1*
**IL-1β**	**i.a.**	0.0 ± 0.4	1.1 ± 0.1	0.3 ± 0.2	0.5 ± 0.8
	**i.v.**	3.8 ± 0.5***	0.2 ± 0.3	4.2 ± 0.3***	1.5 ± 0.5
**Saa1**	**i.a.**	0.3 ± 0.5	1.8 ± 0.4	0.5 ± 0.1	-1.5 ± 1.6
	**i.v.**	2.4 ± 0.3	0.7 ± 0.5	2.7 ± 0.8	2.7 ± 0.5

Gene expression in the liver of arthritic C57Bl/6 mice at day 3 of the AIA model, 2 hours after injection of PVA-SPIONs or PVA alone. Depicted as mean ± SEM, ddCt corrected for PBS injections and GAPDH. n = 3 mice per group,

* = p<0.05,

*** = p<0.0001 when using a one-way ANOVA compared to PBS injected.

**Fig 5:**
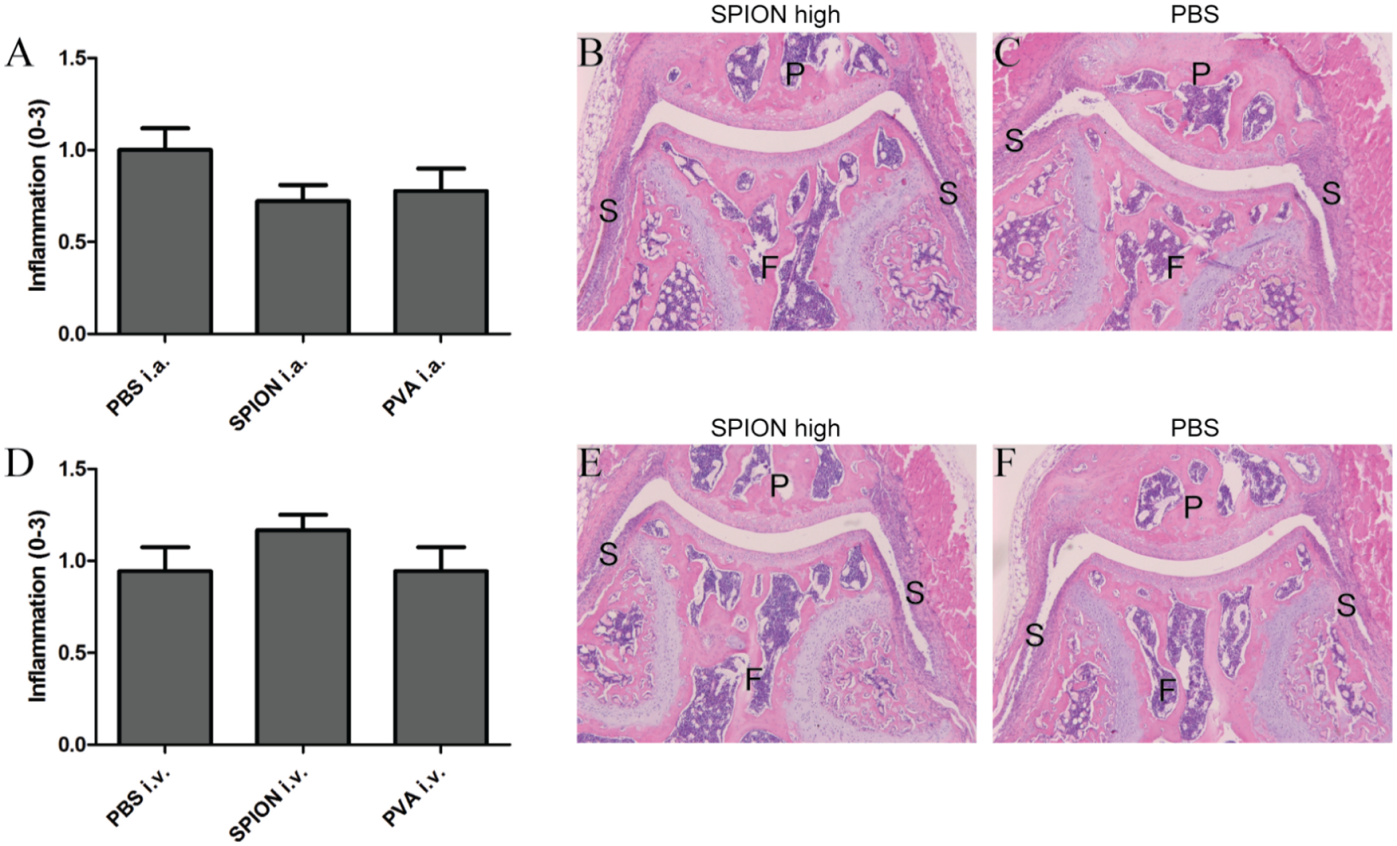
Histological analysis shows no aggravation in joint pathology 14 days after SPION injection. Histological scoring of inflammation in knee joints of arthritic mice, 14 days after i.a. (A) or i.v. (D) injection of a high dose of SPION or PVA. No differences in synovial inflammation are seen between the i.a. SPION (B), the i.v. SPION (E) injected groups and the control i.a. PBS (C) and i.v. PBS (F) injected groups. Magnification 50x. F = femur, P = patella, S = synovium. n = 8 mice per group, one-way ANOVA used.

**Fig 6:**
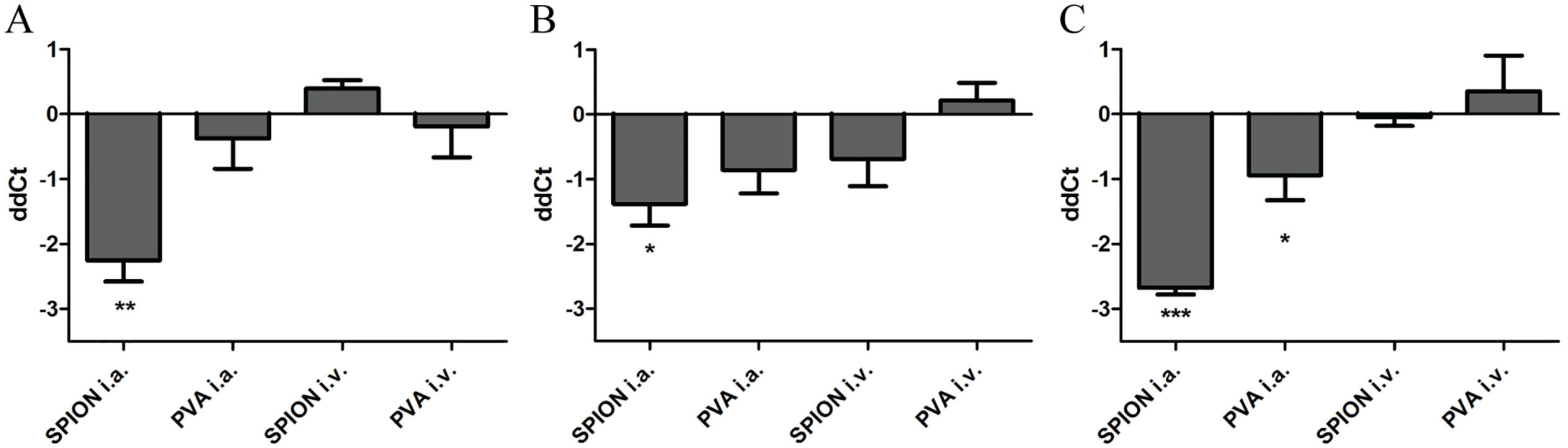
Downregulation of IL-1β gene expression 7, 10 and 14 days after i.a. SPION injection. Gene expression of IL-1β in the liver of arthritic mice, 7 (A), 10 (B) and 14 (C) days after SPION injection (high dose) show a significant downregulation, but only after i.a. injecting of SPION. n = 5 mice per group, * p<0.05, ** p<0.01, ***p<0.05 when using a one-way ANOVA.

Long-term effects, up to 14 days after injection of SPION or PVA, were also investigated using the high dose. After i.a. injection of SPION no adverse consequences were observed on gene expression profiles in the synovium (data not shown). Concomitantly, histology (Fig 5) and serum cytokine concentrations did not show signs towards a change in inflammation. However, when looking at gene expression profiles in the liver, a downregulation of IL-1β was seen at day 7, 10 and 14, but only after i.a. injections (Fig 6). Intravenous injections did not elicit either a pro or anti-inflammatory response in the synovium (Fig 5) or the liver.

## Discussion

The implications of injecting nanoparticles on the severity and course of arthritis has never been studied *in-vivo*, although some *in-vitro* studies point towards an inflammatory effect. Therefore, in this study we investigated the effects SPION may have on arthritis. We have shown that local or systemic injection of SPION causes a short-lasting inflammatory response within 2 days after SPION injection, but 14 days after injection no long-term adverse effects on the course of experimental arthritis were observed.

It was reported previously that PVA-coated SPION can accumulate at sites of inflammation in rat [19]. By using MR imaging, we showed that, when injected i.v., part of the SPION will migrate to the joint, but only when joint inflammation is present. This was also shown previously by Beckmann and co-workers in a study [20], where nanoparticles coated with dextran were injected i.v. in rats with antigen-induced arthritis. Significantly less nanoparticles accumulated in the joints of dexamethasone-treated rats.

We observed that SPION injected i.a. stay in the joint for at least 14 days after injection, in both an inflammatory as well as a non-inflammatory environment. Because SPION apparently reside in the joint for quite some time, it is important to examine any potential inflammatory reactions to these foreign particles. Two different dosages of nanoparticles were used, chosen from a clinical point of view. The low dose did not show any pro-inflammatory effects in naïve as well as arthritic mice. The high dose provoked some short-lasting inflammatory signs, which are discussed below.

In non-arthritic mice, we found a short-lasting pro-inflammatory response of the synovial tissue two hours after the SPION were injected i.a. and also a short-lasting pro-inflammatory response of the liver two hours after the particles were injected i.v. However, this response to the nanoparticles was only transient, since two days after the injection, no inflammatory effect was observed anymore. It is known that SPION can cause oxidative stress in cells [21], which can lead to activation of AP-1 and NFκB and eventually lead to the release of pro-inflammatory cytokines. We indeed found upregulated gene expressions of IL-6 and other pro-inflammatory cytokines, but not of TNFα. Remarkably, gene expression of IL-1β was also significantly elevated in the synovium after i.v. injection, but no signs of inflammation were seen on histology. This increased IL-1β expression can be caused by a mild inflammation of the blood vessel walls in the synovium. Synovial tissue is highly vascularized and nanoparticles may cause an activation of endothelial cells in the blood vessels [22]. An *in-vitro* indication of the pro-inflammatory effects of SPION came from Laskar *et al*. [23]; they incubated M2 macrophages, which are known as the anti-inflammatory subtype, with SPION and found a shift of M2 macrophages towards the more pro-inflammatory macrophage subtype, the M1. Kodali and co-workers [24] also found an impaired ability of macrophages, incubated with SPION, in shifting from M1 to M2.

Whereas different research groups found activation of a variety of cell types after SPION incubation [14, 25], meanwhile other groups found no adverse effects. For instance, Cengelli *et al*. [26] used SPION to explore the option of these particles to be phagocytosed by specific cells in brain-derived structures. Inflammatory activation via nitric oxide of endothelial cells incubated with SPION was investigated, but they did not find higher nitric oxide levels, indicating no inflammatory activation of these cells. Also, when injecting these particles in naïve C57Bl/6 mice no side effects were observed, demonstrating *in-vivo* biocompatibility. This indicates that effects can vary between different sizes and types of SPION and coating, but also between different cell types from different organs.

Although PVA is accepted as a safe polymer to use in humans, most studies are carried out with hydrogel implants coated with PVA [27, 28] and not with PVA in solution. We found, after injection of PVA alone, a short-term inflammatory effect that lasted one day, which was even more severe than the injection of SPION. From this we can conclude that PVA alone causes substantial inflammation, but when PVA is coupled to a nanoparticle it becomes less inflammatory.

Since it is difficult to detect LPS contamination *in-vitro* in nanoparticle samples [29], we used TLR4-/- mice to test for LPS contamination in our experimental *in-vivo* setup. LPS can bind to TLR4, causing a release of pro-inflammatory cytokines as TNFα and IL-1β [30–32]. Upon SPION injection into TLR4-/- mice, similar inflammatory responses were found as in C57BL/6 mice; hence this signaling was not mediated through TLR4, eliminating the possibility of LPS contamination.

Because SPION provoke a pro-inflammatory response in naïve mice, it is also important to know if these nanoparticles aggravate existing inflammation, in this case, arthritis. Therefore, SPION were injected into a highly inflamed knee joint, which again caused a pro-inflammatory response in the synovium two days after SPION injection. However, this response was only detectable on gene expression, and was clearly less severe compared to SPION injection into a naïve knee joint. A similar response was observed in the liver upon systemic administration of nanoparticles: two hours after i.v. injection of SPION in arthritic mice, the liver responded by upregulating TNFα and IL-1β gene expression, but again this was less severe compared to naïve mice. Of clinical importance, these effects were no longer detected at day 7, 10 or 14 after SPION injection and were therefore regarded as transient and short-lasting.

There are also indications that SPION can cause a downregulation of pro-inflammatory genes [24, 33], and a subsequent diminishing of inflammation might therefore be expected. At the later time points of our animal study, we indeed reported lower gene expression levels of IL-1β, but not of TNFα, in the liver after i.v. injection of SPION into arthritic mice. Wu *et al*. [34] also reported a suppressed production of IL-1β, but not TNFα, in LPS stimulated microglia cells, that were subsequently incubated with SPION. However, we did not observe a decrease nor increase in the course of arthritis between SPION injected and PBS-control injected animals at these time points. In addition, no effect was observed on histology or serum cytokine concentrations, even though the SPION were shown to be still present in the arthritic joint as seen on MRI and confirmed by Prussian blue stained histological sections.

## Conclusions

Injection of a high dose of SPION for the MR imaging of joint inflammation causes an inflammatory response that is more pronounced in naïve mice compared to arthritic mice, while the low dose of SPION did not elicit a response. In the long term, up to 14 days after the injections, no adverse effects appear and the injection of SPION does not influence the course of arthritis. Therefore, we conclude that PVA-coated SPION can be used safely to detect joint inflammation by in vivo MR imaging.
